# The Synergy of Double Cross-linking Agents on the Properties of Styrene Butadiene Rubber Foams

**DOI:** 10.1038/srep36931

**Published:** 2016-11-14

**Authors:** Liang Shao, Zhan-You Ji, Jian-Zhong Ma, Chao-Hua Xue, Zhong-Lei Ma, Jing Zhang

**Affiliations:** 1 College of Chemistry and Chemical Engineering, Shaanxi University of Science and Technology, Xi’an 710021, China; 2 Key Laboratory of Chemistry and Technology for Light Chemical Industry, Ministry of Education, Xi’an 710021, China; 3 College of Light Industry and Engineering, Shaanxi University of Science and Technology, Xi’an 710021, China; 4 College of Arts and Sciences, Shaanxi University of Science and Technology, Xi’an 710021, China

## Abstract

Sulfur (S) cross-linking styrene butadiene rubber (SBR) foams show high shrinkage due to the cure reversion, leading to reduced yield and increased processing cost. In this paper, double cross-linking system by S and dicumyl peroxide (DCP) was used to decrease the shrinkage of SBR foams. Most importantly, the synergy of double cross-linking agents was reported for the first time to our knowledge. The cell size and its distribution of SBR foams were investigated by FESEM images, which show the effect of DCP content on the cell structure of the SBR foams. The relationships between shrinkage and crystalline of SBR foams were analyzed by the synergy of double cross-linking agents, which were demonstrated by FTIR, Raman spectra, XRD, DSC and TGA. When the DCP content was 0.6 phr, the SBR foams exhibit excellent physical and mechanical properties such as low density (0.223 g/cm^3^), reduced shrinkage (2.25%) and compression set (10.96%), as well as elevated elongation at break (1.78 × 10^3^%) and tear strength (54.63 N/mm). The results show that these properties are related to the double cross-linking system of SBR foams. Moreover, the double cross-linking SBR foams present high electromagnetic interference (EMI) shielding properties compared with the S cross-linking SBR foams.

Elastic foaming materials are known to exhibit many advantages over unfoamed materials. These advantages include a light weight, higher impact strength^1^, higher fatigue life and cost reduction^2^, higher sound insulation^3^. Because of these excellent properties, they have been used in many applications, including thermal and sound insulators, packaging, electromagnetic shielding materials, structural components and medical devices^4^,^5^. In recent years, elastic foams have been successful made from styrene butadiene rubber (SBR)^6^, poly(ethylene vinyl acetate) (EVA)^7^, poly(ethylene propylene diene) (EPDM)^8^, natural rubber (NR)^9^, polyurethane (PU)^10^ and polychloroprene rubber (CR)^11^. The literature review revealed that the rubber foams were mainly studied from two aspects: (a) their morphology, microstructure and resultant properties; (b) Physical and mechanical properties such as density, hardness, rebound resilience, compression set, tensile strength, tear strength and elongation at break. Our group has studied EVA/polyene elastomer (POE) composite foams^12^, EVA/PU composite foams^13^ and EVA/montmorillonite (MMT) nanocomposite foams^14^. In addition, EPDM foam also was studied by other researchers^15^. The results of all this researches were indicated that the foams have higher compression set (about 30%) and lower elongation at break (less than 600%), which limit its wide application.

Styrene butadiene rubber (SBR) consists of a compound obtained by mixing ~77 percent butadiene and 23 percent styrene as shown in Fig. 1. It has good compression set, enhanced crack resistance, abrasion-resistance, wear resistance, cost reduction, improved aging properties and thermostability^16^,^17^. Some studies enhance SBR by organic fillers such as chitin^18^, lignin^19^, and inorganic fillers^20^,^21^. The results investigate that the mechanical properties and thermal stability of SBR materials increase with the content of fillers. The cross-linking density is also influenced by the structure and surface chemistry of the reinforcing filler^22^,^23^. The effect of peroxide cross-linking on the structure and mechanical properties of poly(styrene-b-butadiene-b-styrene) triblock copolymer(SBS)/polystyrene(PS)/SBR composite foams was investigated by S.R. Sheng *et al*.^24^. Auxetic nanocomposite based on SBR foam with varying nano-carbon loading was studied by some researches^16^.

However, S cross-linking SBR foams show higher shrinkage due to the cure reversion and partial vacuums formed in the closed-cell foam as reaction temperatures dissipate^25^. Shrinkage is also affected by heat transfer, flow-induced crystallization and residual stresses caused by the orientation of molecular chain^26^. Therefore, the fabrication of sulfur cross-linking SBR foam materials is hard to control, and high shrinkage leads to reduced yield and increased production cost.

In this study, SBR foams with lower shrinkage were prepared by introducing the double cross-linking system (sulfur (S) and dicumyl peroxide (DCP)). Most importantly, the synergy of double cross-linking agents was reported for the first time to our knowledge. The shrinkage mechanism was analyzed by the double cross-linking system and the microstructure of SBR foams. Field emission scanning electron microscope (FESEM), Fourier transform infrared spectrum (FTIR), Raman spectra, X-ray diffraction analysis (XRD), thermal gravity analysis (TGA) and differential scanning calorimetry (DSC) were used to characterize SBR foams. The physical and mechanical properties of the foamed samples such as density, shrinkage, compression set, hardness, the tensile strength, elongation at break, tear strength and peel strength were measured. The effect of double cross-linking system on these properties of SBR foams was investigated. Meanwhile, the electromagnetic interference shielding (EMI) and dielectric properties were studied.

## Methods

### Materials

Styrene butadiene rubber (SBR-1502) containing styrene monomer was supplied by the Petro China Co., Ltd. Sulfur (S) and dicumyl peroxide (DCP), used as a cross-linking agent was provided by Tianjin Fuchen Chemical Reagents Factory (Tianjin, China). Azodicarbonamide (AC), used as a foaming agent (decomposition temperature range was 200 ± 3 °C), was supplied by Jinlang Fine Chemical Co., Ltd (Fujian, China). White carbon black (WCB) and solid paraffin (Sp), used as reinforce reagent, were supplied TaiChang resin materials Co., Ltd (Dongwan, China) and Petro China Co., Ltd, respectively. Zinc oxide (ZnO) and Stearic acid (St), used as activators, were supplied by Dongtai Hongyuan Chemical Factory. Tetramethylthiuram disulfide (TMTD), 2,2′-dibenzothiazole disulfide (DM), 3-Diphenylguanidine (D) and N-cyclohexylbenzothiazole-2-sulphenamide (CZ) were supplied by Hebi Uhoo Rubber Chemicals Co., Ltd (Henan, China).

### Preparation of the SBR foams

The raw SBR was prepared in an internal mixer S(X) M-0.5L-K (Suyan, Jiangsu, China, 3 litre, fill factor 0.75 and rotor speed 35 rpm/min) for 15 min at 110 °C. Then the filters, cross-linking agent, foaming agent and accelerators were added into the internal mixer to mix with SBR matrix uniformly. Samples from 1 to 8 of the SBR foams were based on the same composition. The ingredients in the rubber compounds were (in phr): rubber, 100; ZnO, 9; St, 3; WCB: 25; Sp: 5; AC: 3; accelerators (CZ, DM, TMTD, D): 0.167. In addition, the content of cross-linking agent was listed in Table 1. The compounds were taken out from the mill to form a sheet (about 5 mm) by a two-roll mill (XH-401C, Xihua, Guangdong, China) and stored at room temperature for 12 h before foaming. Finally, the foamed sample was obtained by a flat-panel curing press (XH-406, Xihua, Guangdong, China) at a temperature of 200 °C. A higher foaming temperature of 200 °C was chosen in order to decompose the foaming agent adequately to prevent SBR foam from aging.

### Characterization

According to these standard samples, shrinkage should be evaluated in the condition of marking off 10 cm on the surface of the foam samples, and then put them into the oven at 60 °C for 6 h, at which time are taken out from oven and allowed to cool (about 4 h at 20 °C), and the length of line was measured. The shrinkage is calculated by the following equation (1), which was reported by X.G. Deng *et al*.^27^.


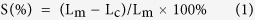


where S is the percentage of shrinkage, L_m_ is the length of the original sample and L_c_ is the length of the final sample after cooled. The density of samples was fast measured by using Electronics Weight Scale (HT-200, A&D, Japan). The hardness (Shore A) of the surface of samples was measured with the Shore A hardness Tester (Xihua, Guangdong, China). The rebound resilience (elasticity) was measured by using the Rebound Resilience Tester (HT-225, Gotech, Taiwan). The heavy hammer was released from a horizontal position, then struck the foamed sample at a vertical point and went back to a certain height. The cross-linking density of the sample was determined using the swelling method^28^. Compression set, defined as the measurement of permanent deformation of foams, was tested by Compression Permanent Deformation Instrument (Xihua, Guangdong, China). The primary thickness of the samples was 10 mm (T_o_). After compression the thickness decreased to 50% (5 mm). After 5 h at 50 °C, the sample was taken out and the final sample thickness (T_f_) was measured and the compression set was calculated using the following equation (2), which was modified from the equation reported by Duck-Ryul Yu^2^.


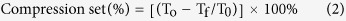


The tensile strength (stress), elongation at break (strain), tear strength and peel strength of the foamed samples were obtained by Desktop Tensile Strength Tester (AI-3000, Gotech, Taiwan.) at room temperature. The speed was 100 mm/min. All measurements were performed three times to get the average results.

TA Instrument Thermogravimetric Analyzer was used for Thermal Gravity Analysis (TGA) and Differential Scanning Calorimetry (DSC). Heating rate was 10 °C/min in the air. The vulcanization characteristic of the SBR foams was determined using a flat die rheometer (BLH-III, Tianfa, Jiangsu, China). Cured sheets of 2 mm in thickness were compression molded at 200 °C. The morphology of the foams was investigated by SEM at 1 kV (S-4800, Hitachi, Japan). The samples were sputter-coated with gold using a vacuum sputter-coater before SEM analysis. SEM studies were performed to characterize the following items: the cell structure. The mean size and distribution of cell were measured by Nano Measurer software. FTIR spectra were recorded on a Bruker VECTOR 22 spectrometer and the all samples were treated as 0.5 mm of the thickness and measured by the attenuated total reflectance method. The Raman scattering experiments were performed using a Renishaw-invia spectrometer. Each spectrum was collected in the frequency range 100–4000 cm^−1^ over 60 s and with 10 accumulations to avoid electronic peaks and average background. The SBR foams Raman data, which up to now are partially reported in the literature, were fully assigned and interpreted. X-ray diffraction (XRD) patterns were recorded in the range of 2θ = 4–60° by step scanning with Ultima IV (RIGAKU, Japan). The electromagnetic shielding and dielectric were investigated by a Pulse vector network analyzer (2NB20, Rohde-Schwarz, Germany).

## Results

### Curing characteristics

**Figure 2:**
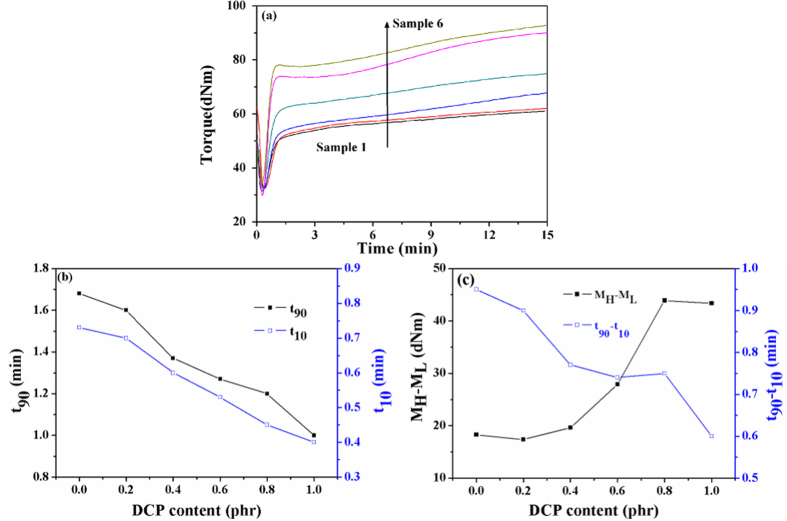
Curves characteristics of SBR foams: (**a**) Rheograph curve; (**b**) The effect of DCP content on scorch time (t_10_) and optimum cure time (t_90_); (**c**) The effect of DCP content on t_90_-t_10_ and M_H_-M_L_.

A typical multichannel data recorder rheograph for the SBR foams is shown in Fig. 2(a). It can be observed that the torque increased with the curing progress of the SBR rubber. Figure 2(b) represents the optimum cure time (t_90_) and the scorch time (t_10_) of various content of DCP. The t_90_ of samples 1 to 6 slowly decreases with increasing DCP content. However, both of t_10_ and t_90_ for sample 2 to 6 have obvious changes with increasing DCP content and they are lower than sample 1. This is because peroxide cross-linking agent system has higher cross-linking efficiency in diene polymer matrix. It is accepted that the difference value (t_90_-t_10_) between t_90_ and t_10_ can be used to evaluate the vulcanization rate, where higher vulcanization rate leads to lower t_90_-t_10_ as shown in Fig. 2(c). In general, the difference value (M_H_-M_L_) between the maximum torque (M_H_) and the minimum torque (M_L_) is related to the cross-linking density^29^. M_H_-M_L_ of the samples increased with the increasing of DCP content as shown in Fig. 2(c). It means the cross-linking density of SBR foams could be increased. Moreover, the physical properties, shrinkage, compression set and other mechanical properties may be enhanced^30^.

In addition, when the DCP content is up to 0.8 phr, the torque shows the dramatically increasing trend after 3 min, which is because the excess DCP content hinders sulfur cross-linking, and results in the sulfur cross-linking time extended. Rheograph curves showed that the synergy of double cross-linking agents was contributed to remit scorch and shrinkage.

### Digital photograph

**Figure 3:**
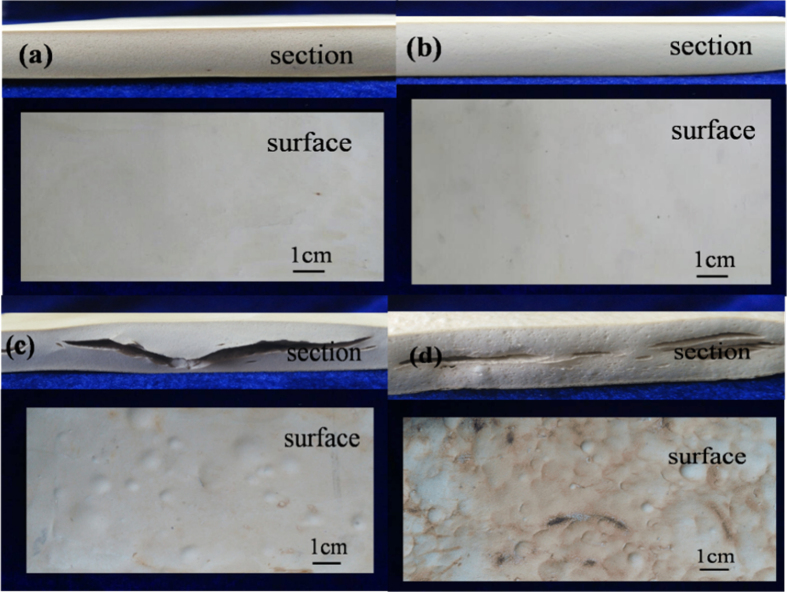
Digital photographs of section and surface of SBR foams: (**a**) sample 1; (**b**) sample 4; (**c**) sample 7; (**d**): sample 8.

Figure 3 shows the digital photograph of section and surface of SBR foams. As shown in Fig. 3(a–d), the surfaces of sample 1 and 4 are smooth, but there are big bubbles on the surface of sample 7 and 8. From the section of samples, the cell size of Fig. 3(a) is bigger than (b). However, the big crack was shown in Fig. 3(c,d). This is because cross-linking rate and the rate of foaming agent decomposing not match with increasing of DCP content^31^. This is because the cross-linking rate is the formation rate C-C or C-S_m_-C bonds of SBR molecular chains in the condition of cross-linking agent exist. In general, the foaming temperature is about 160 °C, but the temperature of cross-linking reaction which induced by DCP is about 120 °C and peroxide cross-linking agent system has higher cross-linking efficiency in diene polymer matrix. In addition, the S cross-ling time was extended by the DCP addition as curing characteristics. It indicates that the DCP content ranges from 0.2 to 1.0 phr. Furthermore, SBR foams cannot be successfully prepared only by DCP as shown in Fig. 3(d).

### Microstructure and morphology

**Figure 4:**
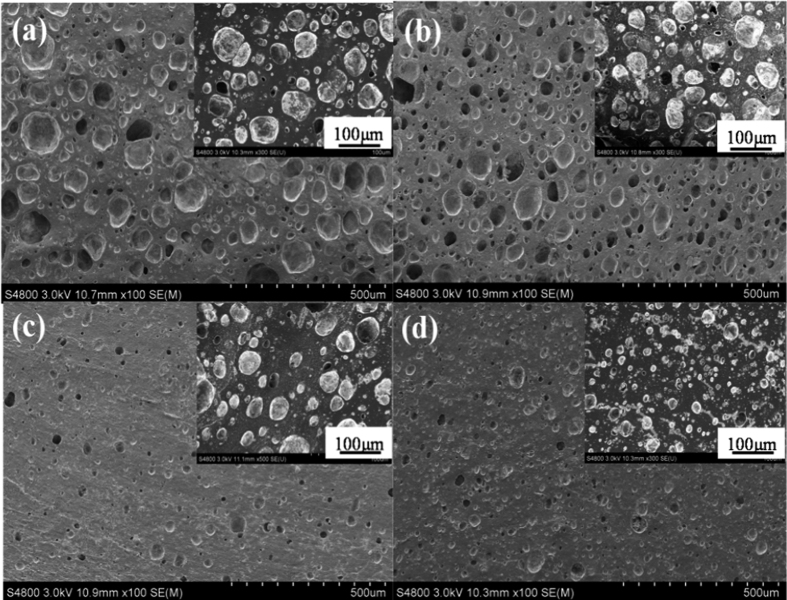
SEM micrographs of surfaces of foams: (**a**) sample 1; (**b**) sample 3; (**c**) sample 4; (**d**) sample 6.

Figure 4(a–d) shows that the cell structure is a mixture of open and closed cells^32^. As shown in Figure S1(a–d), the cell mean sizes (sample 1: 22.94 μm, sample 3: 18.27 μm, sample 4: 12.83 μm, sample 6: 7.68 μm) were decreased with the increasing of DCP content in the SBR matrix from sample 1 to 6. It is because the cross-linking density can be enhanced, which restrict the bubble formation during the vulcanization of the samples^33^. As a consequence, the number of cells increased (as shown in Figure S1) and the cell sizes decreased, which resulted in the change of foam structure. The foam structural variability is related to the mechanical properties.

### FTIR spectra analysis

**Figure 5:**
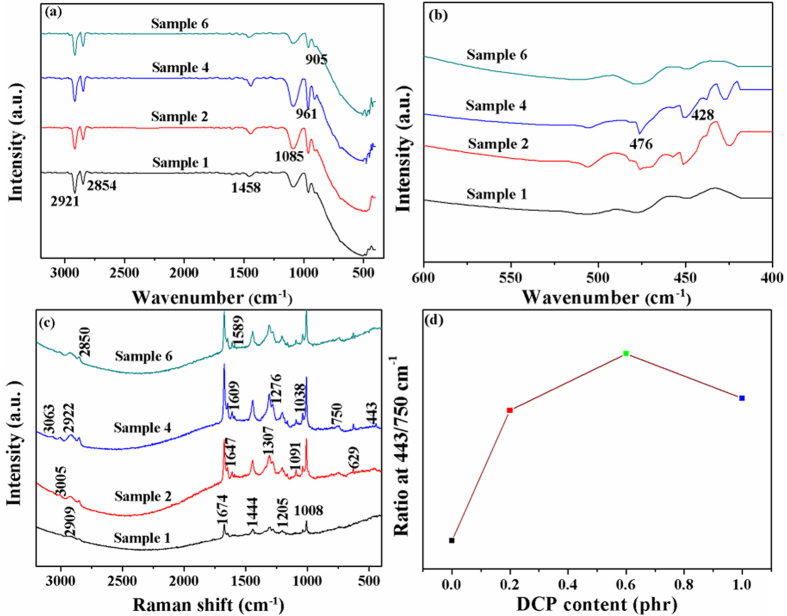
The cross-linking process analysis: (**a**) FTIR spectra of SBR foams (**b**) Local amplification figure of FTIR spectra (**c**) Raman shift of SBR foams (**d**) Ratio at 443/750 cm^−1^.

Figure 5(a) shows the FTIR spectra of sample 1 to 6. The characteristic bands at 2915 and 2854 cm^−1^ are assigned to C-H stretching deformation of -CH_3_ and -CH_2_- in SBR structure unit^34^,^35^. Moreover, with the increasing of DCP content, the intensity of the peaks from sample 1 to 6 is increased, which is related to the decomposition of DCP. As shown in Figure S2, oxygen free radicals formed by heating DCP attack SBR molecular chain to generate radicals of SBR chain and 2-phenylpropan-2-ol, and then the 2-phenylpropan-2-ol turns into acetophenone and prop-l-en-2-ylbenzene in the heating condition. Finally, the cross-linking reaction occurred among radicals of SBR chain. This cross-linking mechanism is in accord with G.L Marshall^36^,^37^.

**Figure 6:**
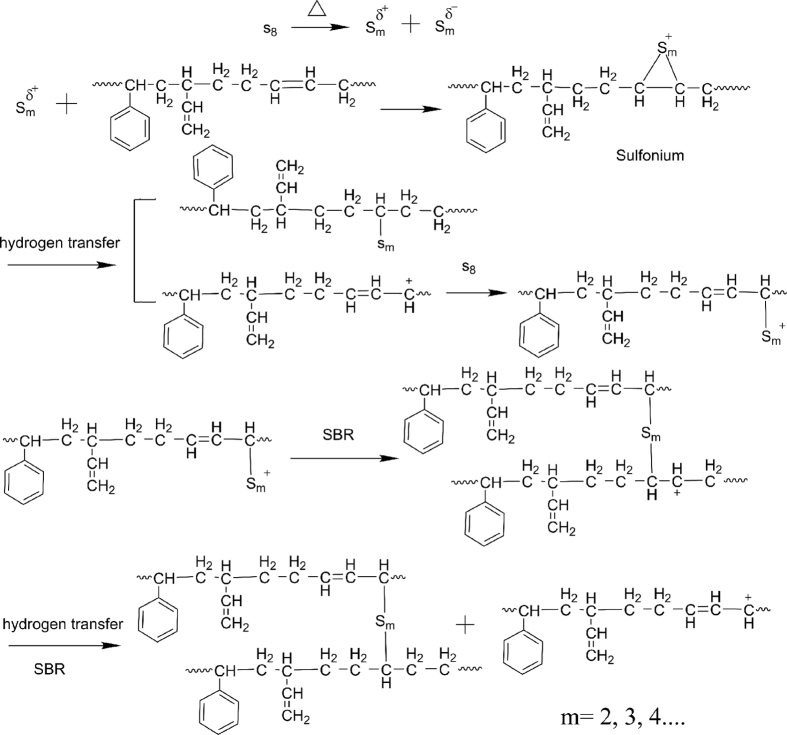
The schematic diagram of reaction mechanism of sulfur with SBR.

The characteristic peak at 1458 cm^−1^ is attributed to -C=C- bending vibration of benzenoid ring. The trans >C=CH- (961 cm^−1^) and the vinyl (>C=CH_2_, 905 cm^−1^) are assigned to the SBR backbone^38^. The characteristic absorption of -C-S- stretching deformation at 1085, 692 cm^−1^ and 476, 428 cm^−1^ (related to -S-S- stretching deformation)^39^ is explained by sulfur cross-linking as shown in Fig. 6. The sulfion by polarized of sulfur reacts with SBR molecule to generate the sulfonium, which reacts with a SBR molecule by hydrogen transfer to produce the polymeric (allylic) carbocation, which undergoes cross-linking by reacting with sulfur followed by addition to a polymer double bond. A subsequent reaction with SBR by hydride transfer regenerates the polymer carbocation. This sulfur cross-linking mechanism is in accord with L. Bateman^40^,^41^.

**Figure 7:**
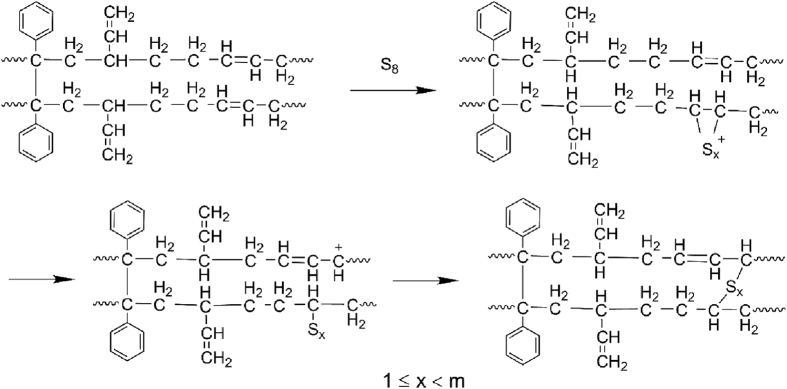
The schematic diagram of double cross-linking mechanism of SBR.

The intensity peaks (at 1085, 476, 428 cm^−1^) increased from sample 1 to 4. Because the sulfur cross-linking rate reaches equilibrium with DCP cross-linking rate, they presented the role of mutual promotion, which was investigated in curing characteristics. With the occurring of DCP cross-linking, the sulfur cross-linking is affected by the decreasing of the distance between molecules. The bond -C-S_m_-C- is difficult to form. Moreover, the number of the bonds -C-S_x_-C- (1 ≤ × < m) increased and the cross-linking density could be enhanced. This is because of the space steric effect of benzene ring and the bonds -C-C- by double cross-linking as shown in Fig. 7. The further quantity of -C-S_x_-C- (1 ≤ × < m) was formed with the occurring of DCP cross-linking, which could benefit the shrinkage and mechanical properties. However, the intensities at 1085, 476, 428 cm^−1^ of the sample 6 (in Fig. 5(a,b)) are decreased, because the bonds -C-C- occurred easily due to the excess of DCP, and the sulfur cross-linking reaction is restrained. This is also investigated in curing characteristics.

Raman spectra of sample 1, sample 2, sample 4 and sample 6 are shown in Fig. 5(c) and band assignments are done based on comparison to literature spectra, as tabulated in Table S1. These Raman bands of SBR foams are in agreement with the literature^8^,^42^,^43^. Both symmetric and asymmetric -CH_2_ and -CH_3_ stretching vibrations typically appear in the 2800–3000 cm^−1^ region. Evidently, -C=C- stretching vibrations of SBR are observed at 1674 and 1647 cm^−1^, respectively. The results showed that intensity of Raman peaks depends on the DCP content. As can be seen, compared with sample 1, the intensities of characteristic signals of samples 2, 4, 6 at 2063, 2922, 1647, 1008 and 629 cm^−1^ tend to increase with the increasing of DCP content. However, as shown in Fig. 5(d), the intensity ratio at 443/750 cm^−1^ increased with the increasing of DCP content, before 0.6 phr, and decreased subsequently. This is because the bonds of -C-S_m_-C- and -S-S- turned into the bonds of -C-S_x_-C- and -C-S- respectively, which is consistent with the FTIR analysis.

### XRD analysis

**Figure 8:**
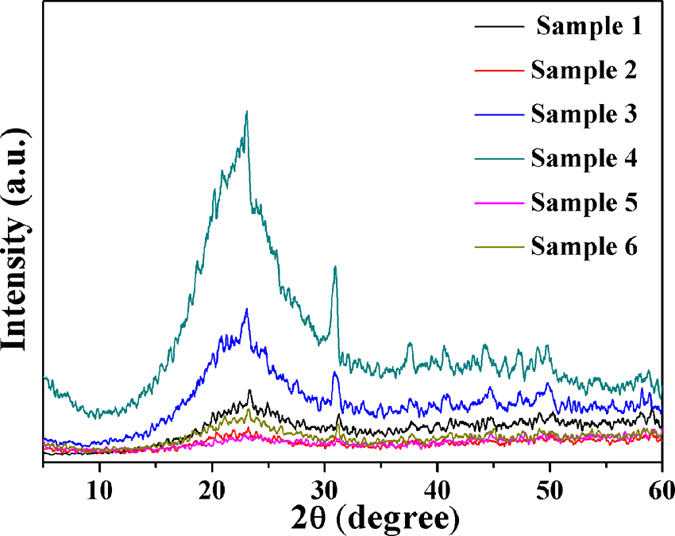
The XRD patterns of samples with different DCP content.

The XRD patterns of samples are shown in Fig. 8. The broad diffraction peak at around 23° can be observed from all the samples. The peak in XRD patterns of sample 4 is much stronger than that of sample 3. This is because the cross-linking density increased with the increasing of DCP content. However, the diffraction peaks of sample 5 and 6 decreased with DCP content, which is because even though the cross-linking density increased, the molecular chains were not easy to crystallize because the curly molecular chain decreased the flexibility of SBR molecular chains, that is, the degree of crystallinity of SBR foams decreased. This is because the shrinkage of the semi-crystalline polymer composites is better than that of the amorphous polymers because of their closely packed structure^44^,^45^.

### DSC analysis

**Figure 9:**
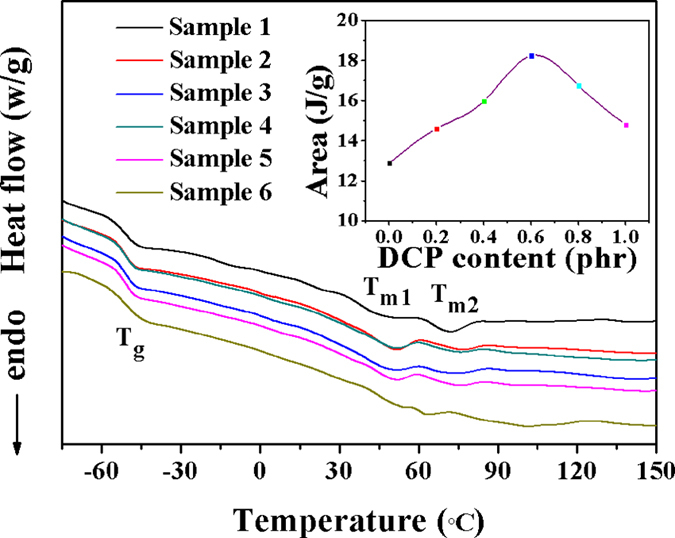
Heat flow curve of samples with different DCP contents.

One stage and two endothermic peaks can be seen on the DSC curves as shown in Fig. 9. The stage at temperature of −50 °C is a consequence of the glass transition temperature (T_g_) and the two endothermic peaks at 50 °C (T_m1_) and 72 °C (T_m2_) are the melt temperature. The double melt peaks are caused by the interior of complete SBR macromolecular crystallization have a lot of the small particles of paracrystal SBR micromolecular in the early stages of the crystallization. As the temperature increases, the small particles gradually disappeared and the second melt peak appeared. There is no obvious change of T_g_ for samples 1–6. The melt peak at lower temperature areas increased from samples 1 to 4 (Fig. 9 inset), indicating that the increasing of crystallinity because the synergy of double cross-linking agents increased the flexibility of SBR molecular chains. The crystallinity of sample 5 and 6 decreased because the exorbitant cross-linking density reduced the athletic ability of SBR molecular chains. This result is consistent with XRD analysis.

### Shrinkage characterization

**Figure 10:**
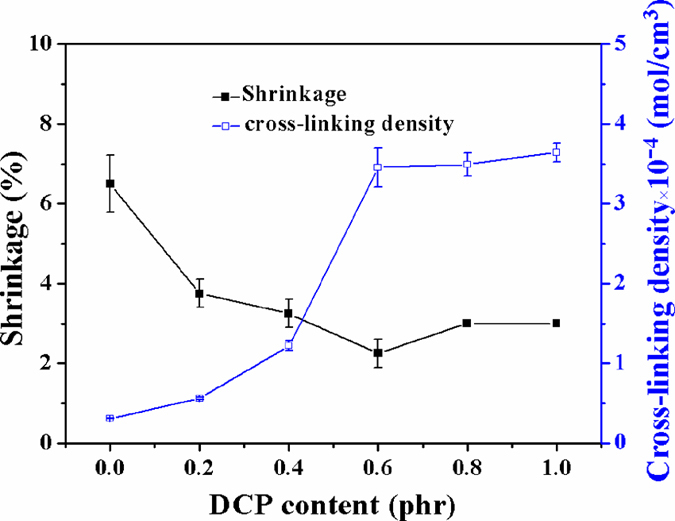
The effect of the DCP content on the shrinkage and cross-linking density of samples.

The effect of DCP content on shrinkage and cross-linking density is shown in Fig. 10. It can be seen that the shrinkage dramatically decreased with the increasing of the DCP content before 0.6 phr from Fig. 10. This is because the cross-linking density (as shown in Fig. 10) and the degree of crystallinity (as shown in XRD and DSC analysis) increased. That is, with the crystallinity of SBR foam increases, the ordered arrangement degree of SBR molecular chains increases. Consequently, the athletic ability of molecular chains decreases, as the gas escapes after foaming, the dimensional stability of the foams is improved and the shrinkage reduces to as low as 2%. This is more excellent than the studies reported as shown in Table 2.

**Figure 11:**
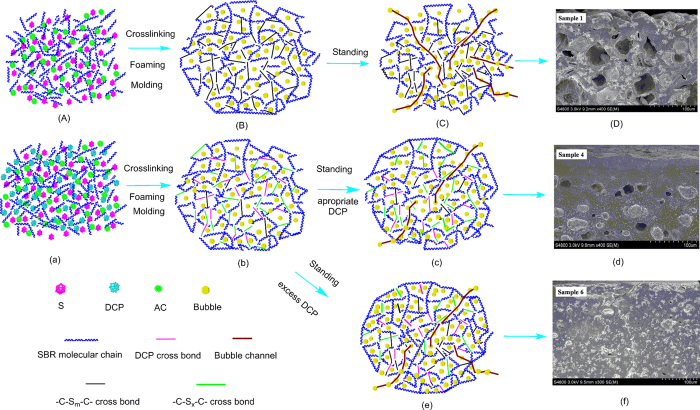
The SBR cross-linking, foaming and shrinkage schematic diagram: (**A**) to (**D**): the sulfur cross-linking agent; (**a**) to (**f**): the double cross-linking agents.

The foaming of SBR and shrinkage schematic diagram of SBR foams are shown in Fig. 11. Sulfur cross-linking process can be described from Fig. 11(A–C). Cure reversion could happen in the samples by single sulfur cross-linking. It means that the network of -S-S- from -C-S_m_-C- in vulcanized rubber was cracked due to the transformation from high curing temperature and long curing time to the surrounding condition^46^. The shrinkage and mechanical properties of SBR foams were decreased. As shown in Fig. 11(C), the foaming gas escapes along the bubble channels. On the one hand, the bubble channels were generated by cure reversion. On the other hand, the bubble channels were formed by the microstructure of SBR foams. As shown in Fig. 11(D) of sample 1, the bigger cell size and the thick wall can be seen because of lower cross-linking density. Furthermore, the skin of the sample is rugged, which results from cure reversion. This indicates that a lot of bubble channels can be produced from the gap among macromolecular chains.

The synergy of double cross-linking agents can be used to describe cross-linking process from Fig. 11(a–c), as shown in FTIR spectra analysis. The formation of -C-C- bond of DCP cross-linking remedies the shortcoming of -S-S- bond fracture, as shown in Fig. 11c. The number of -C-S_x_-C- bonds increased and x < m is resulted in the increasing of cross-linking density, which are contributed to remitting shrinkage and enhance mechanical properties such as tensile strength, tear strength and elongation at break. Compared with the SBR foam of sulfur cross-linking, when the DCP content was 0.6 phr, the smooth and thick skin is formed, as shown in Fig. 11d of sample 4. This microstructure is formed by the synergistic effect of two cross-linking agents and the sulfur cross-linking efficiency was increased by the DCP as shown in curve characteristics (a). The smooth and thick skin is beneficial to hindering foaming gas escape and reduces shrinkage, and the quantity of bubble channels is decreased as shown in Fig. 11d.

With the addition of excess DCP, as shown in Fig. 11(e,f) of sample 6, the number of smaller cells on the skin increased. The space of molecular chain was filled by small cells, which prevent the escaping of foaming gas, compared with SBR foam of simple sulfur cross-linking. However, the overlarge cross-linking density resulted in the incomplete foam of rubber materials, which correspond to the higher density and the low usability.

### Physical and Mechanical properties of the samples

**Figure 12:**
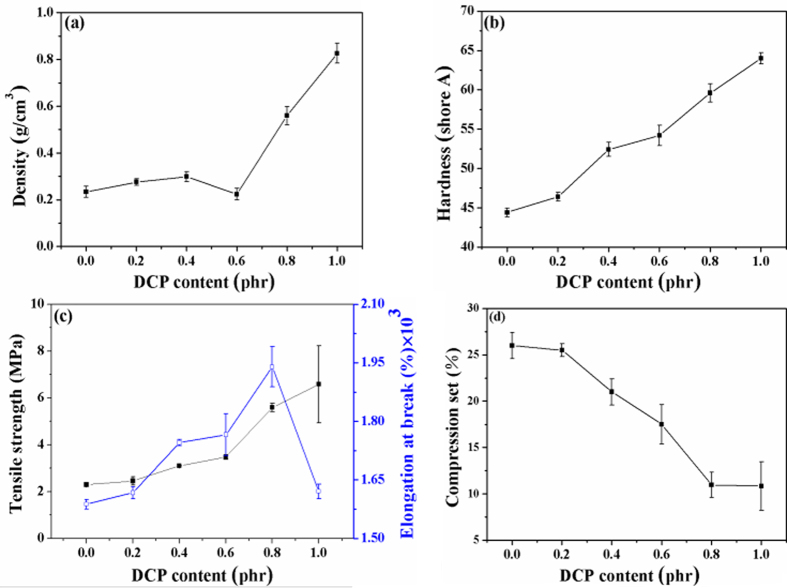
The physical and mechanical properties of SBR foam materials: (**a**) Density; (**b**) Hardness; (**c**) Tensile strength; (**d**) Compression set.

The physical and mechanical properties of samples 1 to 6 are shown in Fig. 12. With the increasing of DCP content from 0.2 to 1.0 phr, the density of SBR foams increased from 0.234 to 0.826 g/cm^3^ (in Fig. 12(a)) due to the increasing of cross-linking density. When the DCP content is 0.6 phr, the foaming gas stored by SBR matrix is the most and the density is the lowest, which is because the cross-linking rate matches with the rate of foaming agent decomposition. As shown in Fig. 12(b), the hardness of the samples increased with the increasing of DCP content due to the synergistic effect of crystallinity and cross-linking density.

The effect of DCP content on the tensile strength and elongation at break of the foams is displayed in Fig. 12(c). The tensile strength is improved with DCP content in the SBR matrix due to the increased cross-linking density. And then, the chemical linkages between SBR chains would improve the resistance to the deformation (the displacement between chains) under the external force. While applying a load, the elongation at break of the foams is based on the extension of soft chains. When the DCP content is lower than 0.8 phr, the elongation at break increased with the increasing of DCP content because of the synergy of double cross-linking agents. When the DCP content is 0.8 phr, the elongation at break reaches up to 1.78 × 10^3^%. The number was far higher than was reported as shown in Table 2. This is contributed to the double cross-linking process. That is, the C-S_m_-C bonds turn into the C-S_x_-C bond with the increasing of DCP content and the number of the C-S_x_-C bond increases. The flexibility SBR chains with a lot of the C-S_x_-C bond offsets the rigidity of SBR chains by added DCP. When the DCP content is more than 0.8 phr, the elongation at break quickly decreased, which is because the excess DCP cross-linking prompt the bond -C-S_m_-C- to turn into -C-S_x_-C-. This result enhances the stiffness of SBR foam. This indicates that the flexibility SBR chains not enough offset the rigidity of SBR chains.

From Fig. 12(d), it can be seen that the compression set of the samples is dramatically reduced with the addition of DCP. The compression set can be decreased to 10% when the content of DCP is 0.8 phr, which was observably higher than was reported as shown in Table 2. It indicates that the SBR foam has good stability when suffered with press and hot. This is because the increasing of cross-linking density of SBR matrix, and then, the stabilization of the cell structure of the foams are improved. The rebound resilience increased in the DCP content is 0.6 phr, as shown in Figure S3(a), due to the synergistic effect of double cross-linking agents. As shown in Figure S3(b), the tear strength increases with the increasing of DCP content, when the DCP content is 0.6 phr, the tear strength is up to 54.6 N/mm.

### Thermal properties

**Figure 13:**
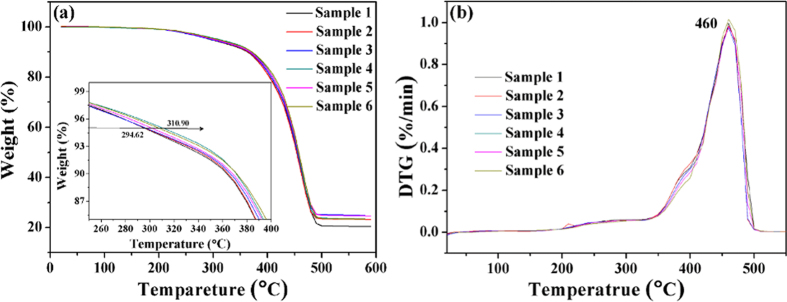
Thermal analysis (**a**): TGA curves of the SBR foams; (**b**): DTG curve of the SBR foams.

The TGA curves of SBR foams are shown in Fig. 13(a). It is obvious that all of the samples had two-step degradation. The first weight loss (the slightly terrace) in the TAG curves at 200–300 °C is attributed to the degradation of unstable additives like the chemical foaming agent, cross-linking agent and other small molecular additives. The second degradation step occurs in the region of 300–500 °C, which is related to the SBR degradation. What is noteworthy is that the thermal stability of samples with double cross-linking is better than sample 1. As shown in the inset of Fig. 13(a), the thermal decomposition temperature increases from 294 to 310 °C, and the difference value is about 15 °C. It can be explained by increasing of the cross-linking density. The corresponding characteristic thermal data of all samples are listed in Table 3. The corresponding weight loss temperatures are increased at the same stages with increasing addition of DCP. The corresponding heat-resistance indices^53^,^54^ of sample 1 to sample 6 are 182.9 °C, 183.6 °C, 185.1 °C, 186.2 °C, 186.7 °C and 188.0 °C, respectively. This suggests that the thermal stability of the SBR foams is improved. The reason is that double cross-linking system contributes to thermal stability. In addition, the DTG curves in Fig. 13(b) showed two peaks that correspond to the local maximum rates of weight loss in all samples. Specifically, the rates of weight loss of samples 2–6 decreased from 390 to 400 °C, which is because the cross-linking density increased with the increasing of DCP content.

### Dielectric properties and electromagnetic interference shielding (EMI) investigations

**Figure 14:**
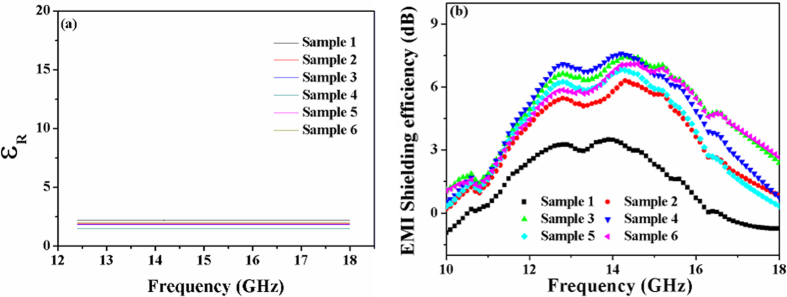
The dielectric (**a**) and electromagnetic interference shielding (**b**) properties of SBR foams.

The dielectric constant (ε_R_) and dielectric loss (ε_I_) of the samples are smaller and similar as shown in Figs 14(a) and S4(a), which means that the composite samples are not conductivity.

In recent years, with the effluence of high frequency electromagnetic wave increasing so rapidly that it becomes a challenge to control it. The SBR foams can provide good electromagnetic shielding properties when the frequency ranges between 12–18 GHz. The overall shielding effectiveness (SE) of a material or shield can be stated as^55^:


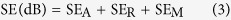


where SE_A_, SE_R_ and SE_M_ are the shielding effectiveness due to absorption, reflection and multiple reflections, respectively. In foams, reflection is related to impedance mismatch between air (in the cells) and absorber (matrix material), absorption is related to energy dissipation in the absorber, and multiple reflections were considered to be due to internal replications that might exist within the material. To understand which mechanisms were operational in the current work, reflection contribution was measured for each sample and was presented in Figure S4(b). The values of reflection shielding effectiveness of all the samples have little changes (−13 ± 1) at about 14 GHz. However, as shown in Fig. 14(b), the samples 2 to 6 revealed larger SE values than that of sample 1 at 12–18 GHz. By comparison, the SE value of sample 4 reached up to 7.5 dB at about 14 GHz. Additionally, absorption mechanism is known to be strongly proportional to electrical conductivity^56^,^57^. Therefore, the overall SE is dominated by multiple reflections rather than reflection and absorption.

Multiple reflections are considered to be due to internal replications that might relate to the cell structure of the foams^58^. As is demonstrated in Fig. 14b, the shielding efficiency of the foams increased with the increasing of DCP content before 0.4 phr, most likely due to the increasing of cell number. The electromagnetic waves have been decreased by the cells in the form of multiple reflections. But the shielding efficiency decreased after 0.4 phr, which may be due to the increasing of hardness (as shown in Fig. 12b) of the foams.

In general, the dielectric constant is higher (the good conductivity). The EMI shielding effectiveness is better. However, the opposite result was obtained in this work, because of the structure changes of the SBR foams. Electromagnetic shielding materials caused by structure may deserve further study.

## Discussion

In summary, the double cross-linking agents, dicumyl peroxide (DCP) and sulfur (S), have a great effect on the shrinkage, size distribution of hollow cells and the results of physical and mechanical properties. Characterizations reveal that the shrinkage was reduced to 2% for sample 4 with the 0.6 phr of DCP content compared with other samples with the same content of foaming agent. XRD has evidenced that the shrinkage depends on the degree of crystallinity. The density, the hardness, shrinkage, tensile strength, tear strength can improve with the increase of the DCP concentration. In addition, the elongation at break reaches up to 1.78 × 10^3^%, and compression set decreases up to 10.96%. Those are much lower than the traditional foaming materials. Also, the size and number of cells have an effect on the electromagnetic interference shielding properties.

## Additional Information

**How to cite this article**: Shao, L. *et al*. The Synergy of Double Cross-linking Agents on the Properties of Styrene Butadiene Rubber Foams. *Sci. Rep.* **6**, 36931; doi: 10.1038/srep36931 (2016).

**Publisher’s note**: Springer Nature remains neutral with regard to jurisdictional claims in published maps and institutional affiliations.

## Supplementary Material

Supplementary Information

## Figures and Tables

**Figure 1:**
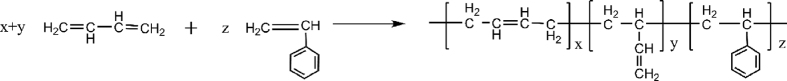
Molecular structure of SBR.

**Table 1:** The loading of cross-linking agent for all samples.

Sample	1	2	3	4	5	6	7	8
DCP	0	0.2	0.4	0.6	0.8	1.0	1.2	0.2
S					0.5			0

**Table 2:** Comparison of physical and mechanical properties between SBR foams and other foam materials reported recently.

Material	Density (g/cm^3^)	Shrinkage (%)	Compression (%)	Strength		Elongation (%)	Reference
				Tensile (M Pa)	Tear (N/mm)		
EVA/MWCNT^a^	0.15	—	40	3.83	15.4	251	47
EVA/POE	0.187	—	31	2.66	—	268.05	48
EVA/CPE^b^	0.12	7.2	—	1.05	4.87	200	49
EVA/TPU	0.151	—	31.8	2.5	5.7	244	50
BR/SBR/NR	—	—	—	11.1	36.3	538	30
EPDM	0.29	—	26	2.44	48.4	626	51
CPE	—	—	15.3	1.93	7.31	269.4	52
SBS/SBR/PS	0.2	5.0	33	0.92	3.2	270	24
SBR	0.223	2.25	10.96	3.475	54.63	1.78×10^3^	This work

^a^Multiwalled carbon nanotube (MWCNT).

^b^Chlorinated polyethylene rubber (CPE).

**Table 3:** Thermal data of the SBR foams from TGA analysis.

Samples	Temperature/°C		Heat-resistance index^a^/°C
	T_5_	T_30_	
Sample 1	294.2	426.2	182.9
Sample 2	296.5	426.8	183.6
Sample 3	301.4	428.5	185.1
Sample 4	304.6	430.1	186.2
Sample 5	307.4	430.0	186.7
Sample 6	310.9	432.2	188.0

^a^Heat resistance index = 0.49 [T_5_ + 0.6(T_30_ − T_5_)]. T_5_, T_30_ is the decomposing temperature at 5%, 30% weight loss, respectively.
