# Erratum: The Synergy of Double Cross-linking Agents on the Properties of Styrene Butadiene Rubber Foams

**DOI:** 10.1038/srep40930

**Published:** 2017-01-25

**Authors:** Liang Shao, Zhan-You Ji, Jian-Zhong Ma, Chao-Hua Xue, Zhong-Lei Ma, Jing Zhang

Scientific Reports 6: Article number: 3693110.1038/srep36931; published online: 11 14 2016; updated: 01 25 2017

This Article contains typographical errors.

In Table 1, information for Sample S has been omitted for columns 1, 2, 3, 4, 6 and 7. These should all read ‘0.5’. The correct table appears below.

In Equation (2),


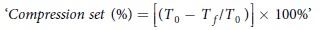


should read


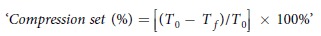


In the Methods section,

‘The primary thickness of the samples was 10 mm (T_o_).’

should read:

‘The primary thickness of the samples was 10 mm (T_0_).’

## Figures and Tables

**Table 1:** 

Sample	1	2	3	4	5	6	7	8
DCP	0	0.2	0.4	0.6	0.8	1.0	1.2	0.2
S	0.5	0.5	0.5	0.5	0.5	0.5	0.5	0

