# Supplementary material for: The Synergy of Double Cross-linking Agents on the Properties of Styrene Butadiene Rubber Foams
Source: Sci Rep. 2016 Nov 14;6:36931. doi: 10.1038/srep36931 (PMC5107997; doi:10.1038/srep36931)
Supplement: Supplementary Information [file srep36931-s1.doc]

**The Synergy of double Cross-linking Agents on the Properties of Styrene Butadiene Rubber Foams**

**Liang Shao,^*,1^,** **^3^ Zhan-you Ji,^1^ Jian-Zhong Ma,^*, 2^ Chao-Hua Xue ^2^ Zhong-Lei, Ma ^1^, Jing Zhang ^4^**

^1^ College of Chemistry and Chemical Engineering, Shaanxi University of Science and Technology, Xi’an 710021, China

^2^ College of College of Light Industry and Engineering, Shaanxi University of Science and Technology, Xi’an 710021, China

^3^ Key Laboratory of Chemistry and Technology for Light Chemical Industry, Ministry of Education, Xi’an 710021, China

4 College of Arts and Sciences, Shaanxi University of Science and Technology, Xi’an 710021, China

^*^Corresponding Author: [shaoliang@sust.edu.cn;](mailto:shaoliang@sust.edu.cn;)[majz@sust.edu.cn](mailto:majz@sust.edu.cn)

**
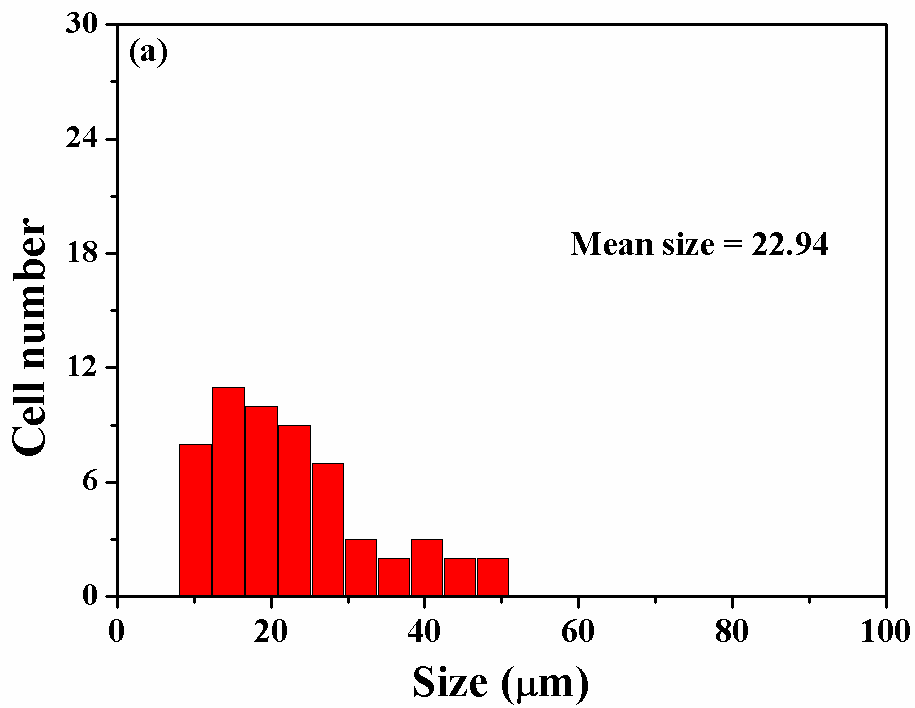

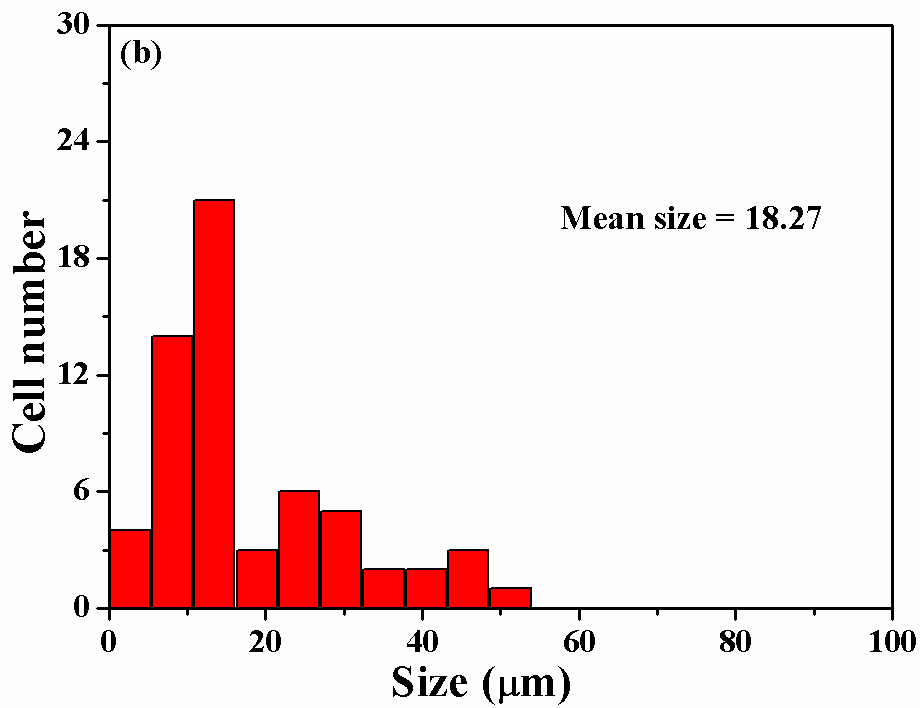

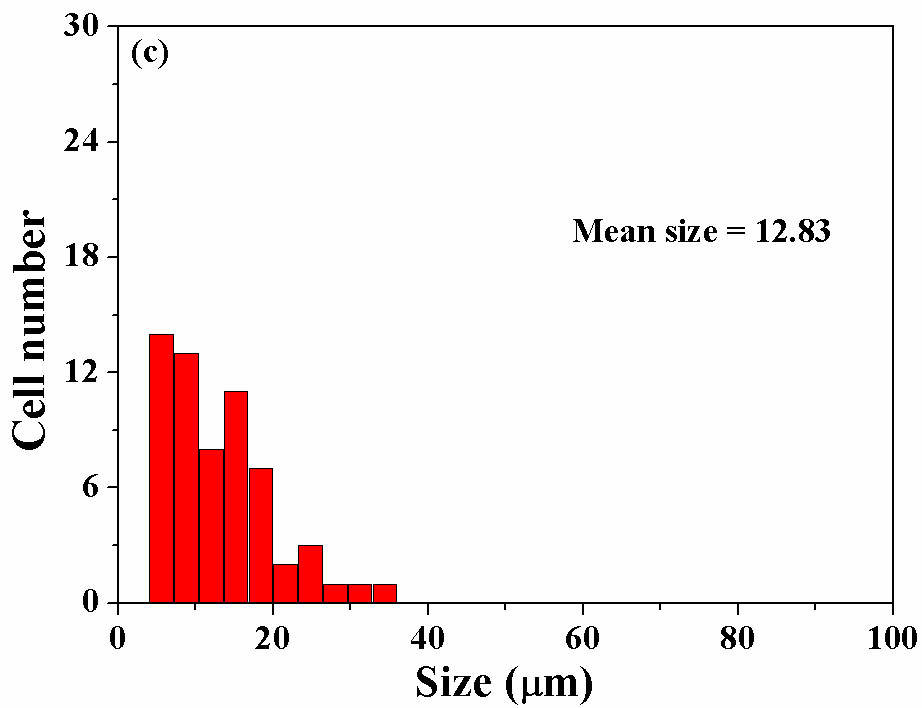

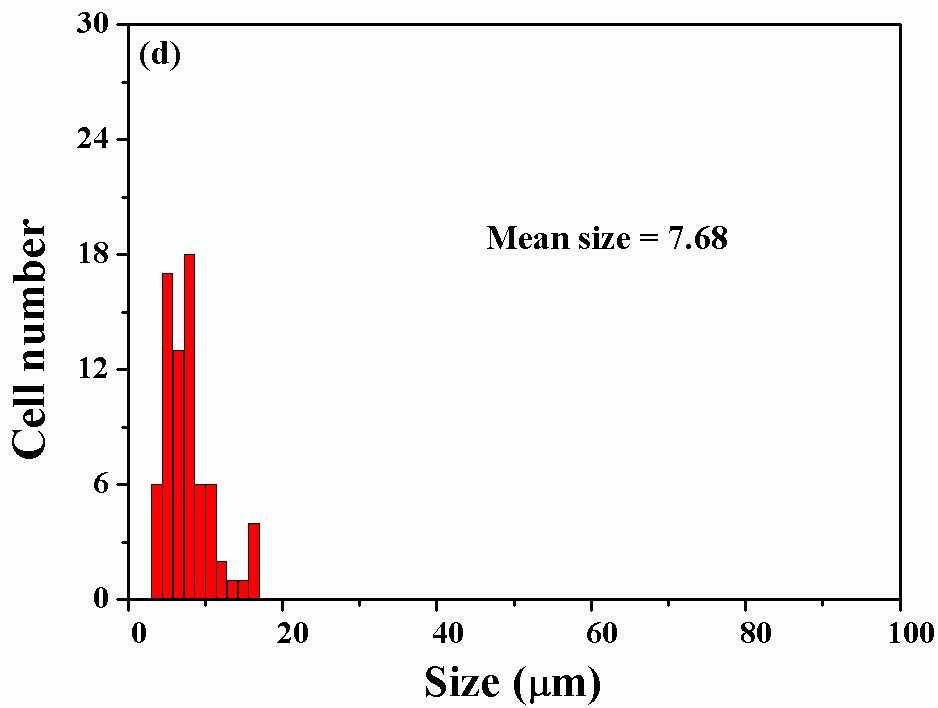
**

Figure S1. Histograms of measured cell diameter of SBR foams with different DCP contents obtained from high magnificent micrographs images: (a) sample1; (b) sample3; (c) sample4; (d)sample6.


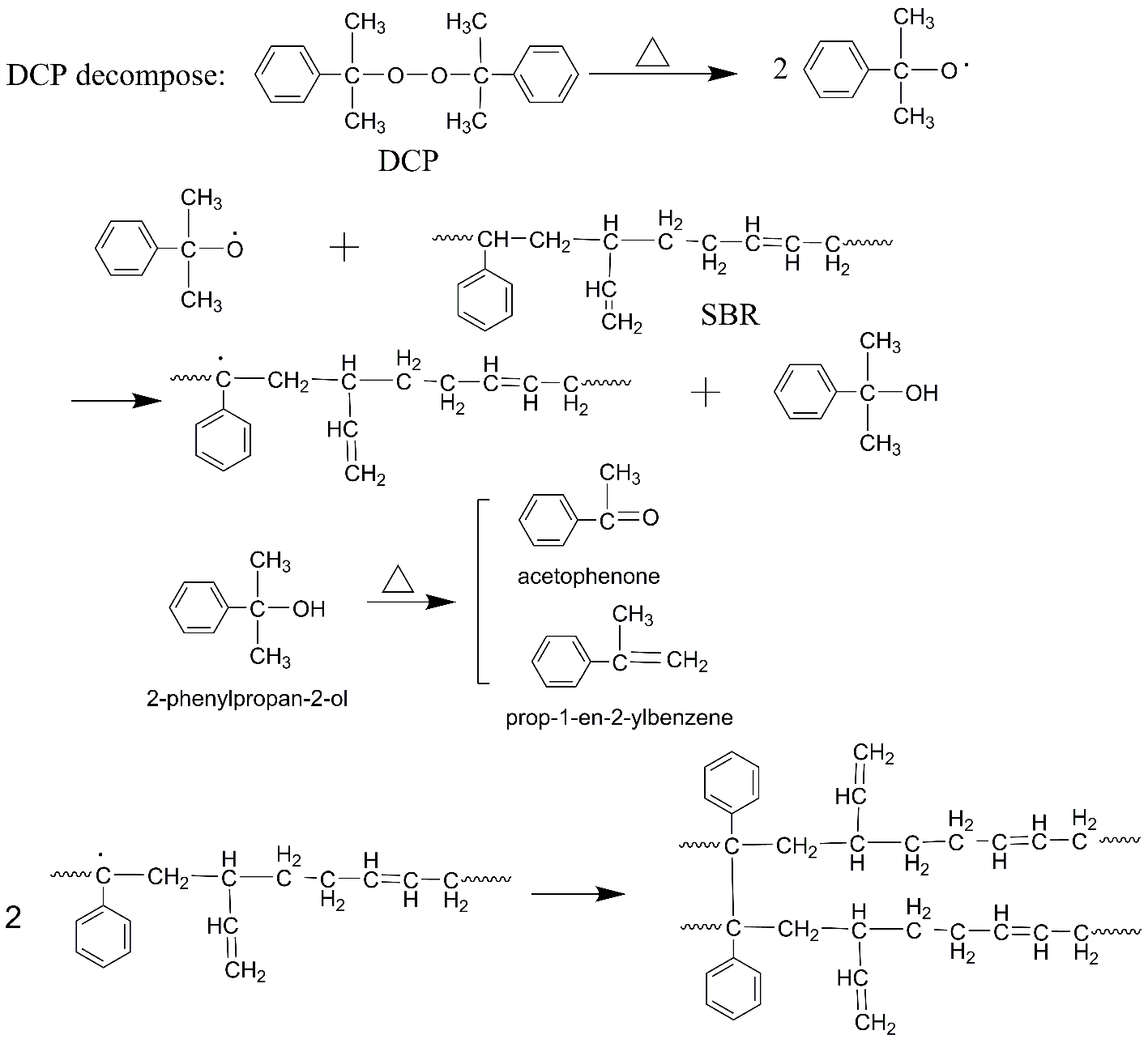


Figure S2 The schematic diagram of reaction mechanism of DCP with SBR.

Table S1 Positions and assignments of bands observed in Raman spectra of the samples.

| Raman shift (cm^-1^) | | | | | Assignment |
| --- | --- | --- | --- | --- | --- |
| a | b |  | c | d |  |
| Literature | This work |  | Literature | This work |
| 3058 | 3063 |  | 3058 | 3063 | =C-H ring stretching |
| 3000 | 3005 |  | 3000 | 3005 | =C-H stretching |
| 2910 | 2909 |  | 2910 | 2922 | CH_2_ asymmetric stretching |
| 2845 | 2850 |  | 2845 | 2850 | CH_2_ symmetric stretching |
| 1668 | 1674 |  | 1668 | 1674 | C=C stretching |
| 1642 | 1647 |  | 1642 | 1647 | C=C stretching |
| 1602 | 1609 |  | 1602 | 1609 | =C-H ring stretching |
| 1582 | 1589 |  | 1582 | 1589 | =C-H ring stretching |
| 1437 | 1444 |  | 1437 | 1444 | CH_2_ deformation |
| 1302 | 1307 |  | 1302 | 1307 | =C-H in-plane |
| 1273 | 1276 |  | 1273 | 1276 | CH_2_ twisting |
| 1199 | 1205 |  | 1199 | 1205 | Symmetric stretching of trans C=C |
| 1094 | 1091 |  | 1094 | 1091 | CH_2_ wagging |
| 1039^[42]^ | 1038 |  | 1039^[42]^ | 1038 | CH_3_ rocking |
| 1002 | 1008 |  | 1002 | 1008 | Symmetric ring breathing |
| 756^[8]^ | 750 |  | 756^[8]^ | 750 | S-S stretching |
| 622 | 629 |  | 622 | 629 | C=CH ring bending |
| 442 | 443 |  | 442 | 443 | C-S stretching |







Figure S3. The physical and mechanical properties of SBR foam materials:(a) Rebound resilience; (b) Tear strength.


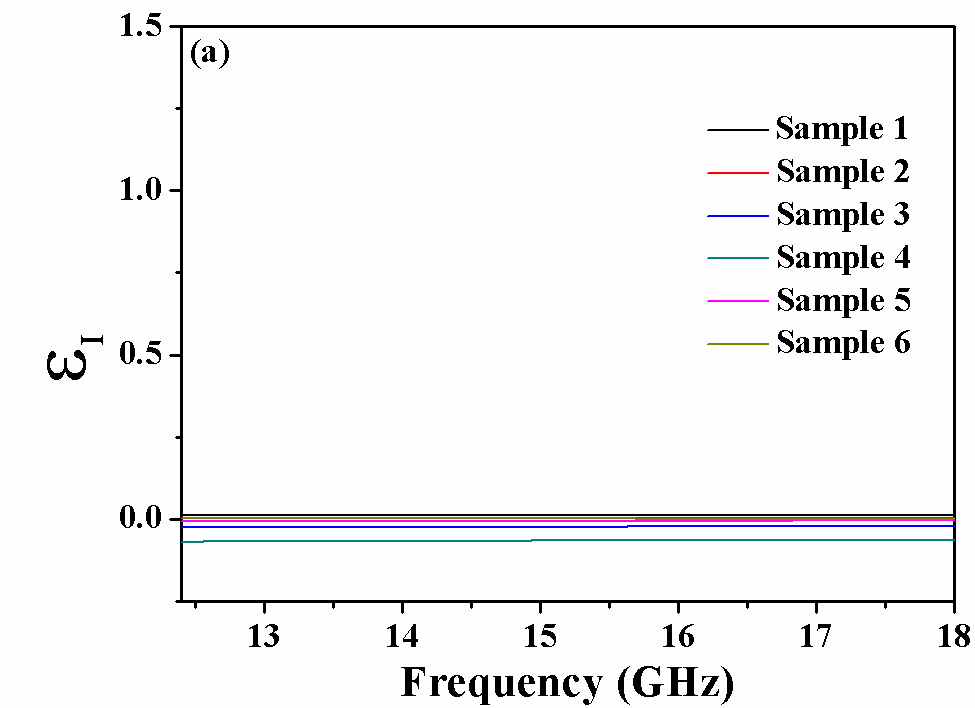

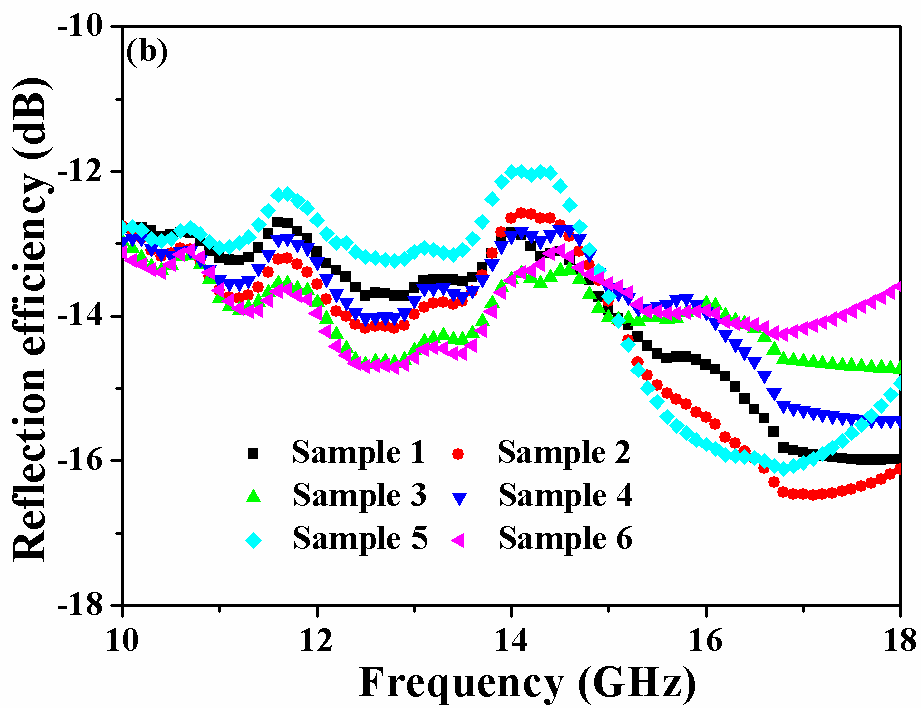


Figure S4. The dielectric properties (a) and electromagnetic interference shielding (b) of SBR foams.
